# Knockout of the polysialyltransferases ST8SiaII and ST8SiaIV leads to a dilatation of rete testis during postnatal development

**DOI:** 10.3389/fphys.2023.1240296

**Published:** 2023-07-14

**Authors:** Luisa Humpfle, Nadim E. Hachem, Peter Simon, Birgit Weinhold, Sebastian P. Galuska, Ralf Middendorff

**Affiliations:** ^1^ Institute of Anatomy and Cell Biology, Medical Faculty, Justus-Liebig-University, Giessen, Germany; ^2^ Institute of Biochemistry, Medical Faculty, Justus-Liebig-University, Giessen, Germany; ^3^ Institute of Cellular Chemistry, Hannover Medical School, Hannover, Germany; ^4^ Institute for Farm Animal Biology (FBN), Dummerstorf, Germany

**Keywords:** polysialic acid, rete testis, testis, epididymis, smooth muscle cells, postnatal development

## Abstract

Polysialic acid (polySia) is a carbohydrate polymer that modulates several cellular processes, such as migration, proliferation and differentiation processes. In the brain, its essential impact during postnatal development is well known. However, in most other polySia positive organs, only its localization has been described so far. For instance, in the murine epididymis, smooth muscle cells of the epididymal duct are polysialylated during the first 2 weeks of postnatal development. To understand the role of polySia during the development of the epididymis, the consequences of its loss were investigated in postnatal polySia knockout mice. As expected, no polysialylation was visible in the absence of the polysialyltransferases ST8SiaII and ST8SiaIV. Interestingly, cGMP-dependent protein kinase I (PGK1), which is essentially involved in smooth muscle cell relaxation, was not detectable in peritubular smooth muscle cells when tissue sections of polySia knockout mice were analyzed by immunohistochemistry. In contrast to this signaling molecule, the structural proteins smooth muscle actin (SMA) and calponin were expressed. As shown before, in the duct system of the testis, even the expression of these structural proteins was impaired due to the loss of polySia. We now found that the rete testis, connecting the duct system of the testis and epididymis, was extensively dilated. The obtained data suggest that less differentiated smooth muscle cells of the testis and epididymis result in disturbed contractility and thus, fluid transport within the duct system visible in the enlarged rete testis.

## 1 Introduction

All cells, both pro- and eukaryotic cells, are surrounded by a glycocalyx (Varki, 2017; Awofiranye et al., 2022). These glycoconjugates are essential for interaction and communication processes between cells, such as the recognition of endogenous and exogenous cells by immune cells. Receptors of immune cells can distinguish between glycan-based pathogen-associated molecular patterns (PAMPs), danger-associated molecular patterns (DAMPs) and self-associated molecular patterns (SAMPs) (Gagneux et al., 2015; Tecle and Gagneux, 2015; Springer and Gagneux, 2016; Bornhofft et al., 2018; Bornhöfft and Galuska, 2019; Varki, 2020). However, the proliferation and differentiation processes of endogenous cells are also regulated by glycoconjugates. For instance, glycosaminoglycans of the glycocalyx, such as heparin, bind growth factors and influence recognition by their growth factor receptors and thus, cellular activation (Wang et al., 2017; Wigén et al., 2019).

Another acidic carbohydrate polymer, polysialic acid (polySia), can act in a similar way. In mammals, polySia is a homopolymer of the sialic acid N-actylneuraminic acid (Neu5Ac) and can be synthesized by two different polysialyltransferases, ST8SiaII and ST8SiaIV (Colley et al., 2014; Schnaar et al., 2014; Galuska et al., 2017; Sato and Kitajima, 2020). Similar to glycosaminoglycans, polySia binds growth factors and influences their activation capacity (Kanato et al., 2008; Ono et al., 2012; Hane et al., 2015; Strubl et al., 2018). Moreover, the negative charge of polySia modulates the adhesion of cells, which additionally affects migration, proliferation and differentiation processes, which take place mainly during the development of the brain (Bruses and Rutishauser, 2001; Kleene and Schachner, 2004; Rutishauser, 2008; Sato and Kitajima, 2013; Schnaar et al., 2014; Galuska et al., 2015). Remarkably, the loss of polySia leads to several neurodevelopmental defects in the brain and a lethal phenotype in knockout mice, demonstrating the essential role of polySia (Weinhold et al., 2005). However, besides the brain, polySia is present in several other organs, such as the testis and epididymis, during postnatal development (Colley et al., 2014; Schnaar et al., 2014; Zlatina et al., 2017; Guo et al., 2019). In contrast to the brain, the impact of polySia during the development of these organs is mostly unknown.

We have recently investigated the postnatal testes of polySia knockout mice to determine the consequences of its loss (Hachem et al., 2021). In the postnatal testis of mice, polySia is mainly located in the peritubular layers of smooth muscle cells of seminiferous tubules, which surrounds the germinal epithelium, and the tunica albuginea encapsulating the whole testis. In adult mice, contractile peritubular cells are involved in sperm transport. Remarkably, in the postnatal testis of polySia knockout mice, peritubular smooth muscle cells developed toward a synthetic phenotype (Hachem et al., 2021). In addition to an increased proliferation rate, proteins of the contractile system seem to be absent. Both the cGMP-dependent protein kinase PKG1, which regulates the relaxation of smooth muscle cells (Ayoubi et al., 2017; Zhang et al., 2017) and the actin-associated protein calponin were not detectable in peritubular smooth muscle cells, whereas the expression of both proteins was not influenced in vascular smooth muscle cells of the postnatal testis. The results suggest that the contraction of peritubular smooth muscle cells is impaired.

Intriguingly, polySia is also present in the smooth muscle cell layer surrounding the epithelium of the epididymal duct (Simon et al., 2015). With increasing physiological collagenization of the contractile peritubular layer, polySia levels decrease in the postnatal epididymis. To characterize the impact of polySia during the postnatal development of the epididymis, tissue sections of different polysialyltransferase knockout mice were investigated. Furthermore, the rete testis, as the connecting element between the tubular systems of the testis and epididymis, was included in the outlined study.

## 2 Material and methods

### 2.1 Animals

Wild-type and knockout mice were housed in the animal facility of the MHH under specific pathogen–free conditions. All protocols for animal use were in compliance with the German law for protection of animals and approved by the local authorities (33.12-42502-04-18/2932). For the outlined study, testicular and epididymal tissue from 5 wild-type (*st8sia2* ^*+/+*^; *st8sia4* ^*+/+*^), 3 *st8sia2* ^*+/−*^; *st8sia4* ^*−/−*^ and 5 double knockout mice (*st8sia2* ^*−/−*^; *st8sia4* ^*−/−*^) (age 9.5 days) were used. In addition, testis and epididymis of postnatal day 7.5 of *st8sia2* ^*+/−*^; *st8sia4* ^*−/−*^ mice were characterized. Some of the tissues have already been used in previous studies (Simon et al., 2015; Hachem et al., 2021).

### 2.2 Histology and immunohistochemistry

The tissues were fixed in Bouin solution as described previously (Simon et al., 2015; Hachem et al., 2021). The fixed tissue was embedded in paraffin, and 5 µm slides were dewaxed and rehydrated for the different staining techniques. The following primary antibodies were used for immunohistochemistry: mouse anti-α-smooth muscle actin (SMA) mAb (Sigma-Aldrich, St. Louis, MO, United States; 2 μg/mL), rabbit anti-calponin mAb (Abcam, Cambridge, United Kingdom; 0.134 μg/mL), rabbit anti-proliferating cell nuclear antigen (PCNA) mAb (Abcam, Cambridge, United Kingdom; 0.378 μg/mL), rabbit anti-PKG polyclonal antibody (pAb) (Enzo, Lausen, Switzerland; 2 μg/mL), and anti-polySia mAb735 (10 μg/mL). The monoclonal antibody (mAb) 735 was provided by Martina Mühlenhoff (Hannover Medical School [MHH], Germany) (Frosch et al., 1985). All primary antibodies were applied to the slides overnight at 4°C. Subsequently, the sections were washed with PBS, and a peroxidase polymer kit (DAKO, Hamburg, Germany) using 3,3′-diaminobenzidin (DAB) as the staining reagent was added to visualize the respective antigens. Nuclei of tissue sections, which were stained for polySia, were visualized with hematoxylin. In addition to immunohistochemistry, AZAN trichrome staining was used (Volkmann et al., 2011). Zeiss Axioskop 2 plus was used to take photos of the stained tissue slides (Carl Zeiss Vision, Munich, Germany). The pictures were processed with Axio Vison Software (Carl Zeiss Vision).

## 3 Results

### 3.1 The deletion of *st8sia2* and *st8sia4* leads to a loss of polySia in the epididymis

**FIGURE 1:**
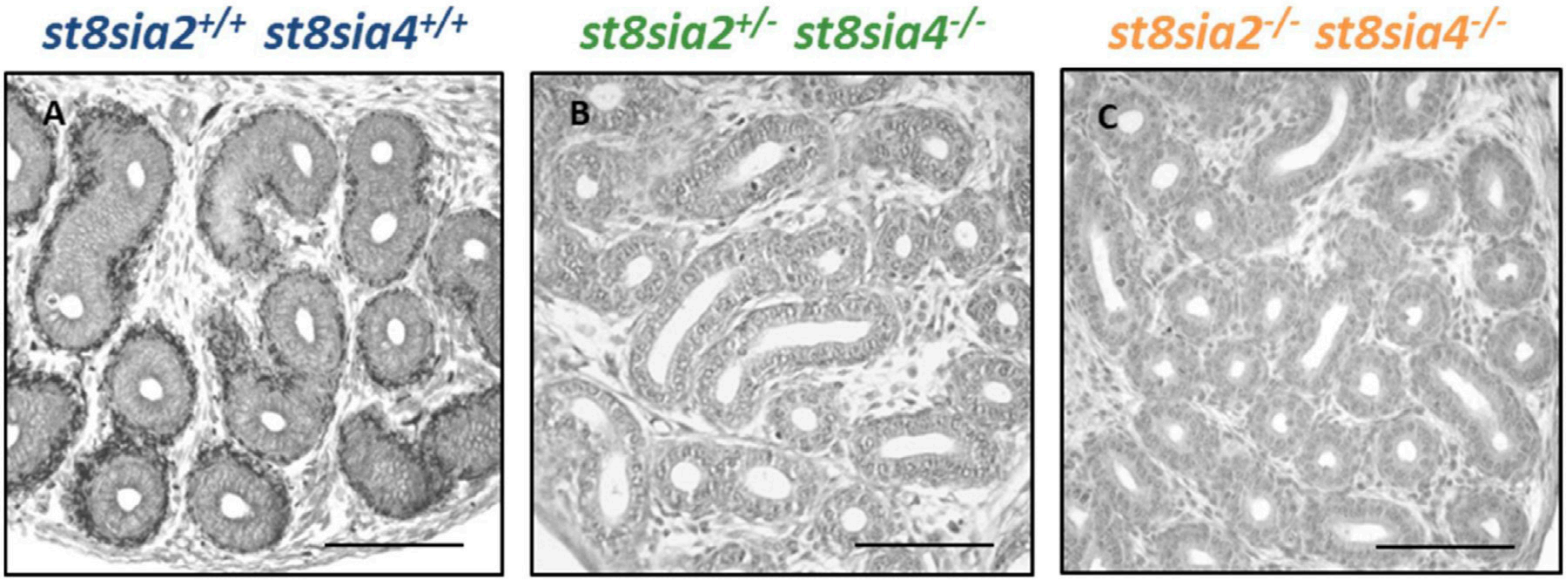
Visualization of polySia in the postnatal epididymis of 9-day-old wild-type and knockout mice. For the immunohistochemical localization of polySia in **(A)** wild-type (*st8sia2* ^*+/+*^; *st8sia4* ^*+/+*^), **(B)** *st8sia2* ^*+/−*^; *st8sia4* ^*−/−*^, and **(C)** polysialyltransferase-deficient (*st8sia2* ^*−/−*^; *st8sia4* ^*−/−*^) mice, mAb 735 was applied. Nuclei were stained with hematoxylin. Scale bars: 100 μm.

During the first 10 days of postnatal development, polySia is mainly localized in populations of smooth muscle cells in the epididymis (Simon et al., 2015). With an increase in collagen synthesis, the polysialylation status rapidly decreases, and two to 3 weeks after birth, smooth muscle cells show no more polySia staining. Based on these results, we analyzed the epididymis of 9-day-old polysialyltransferase knockout mice by immunohistochemistry to control whether the deletion of *st8sia2* and *st8sia4* leads to a loss of polySia. Simultaneously, we analyzed epididymal tissue of wild-type mice. As described in Simon *et al.*, smooth muscle cell populations, which surround the epithelial layer, were polySia positive in wild-type mice (Figure 1). In contrast, no polySia signals were detectable in polysialyltransferase-deficient mice (*st8sia2* ^*−/−*^; *st8sia4* ^*−/−*^), demonstrating that without ST8SiaII and IV, no polySia is produced in the epididymis. Even in mice with one remaining active allele of a polysialyltransferase (*st8sia2* ^*+/−*^; *st8sia4* ^*−/−*^), smooth muscle cells exhibited no polySia staining.

### 3.2 The loss of polySia shows no impact on the expression of smooth muscle actin and calponin in smooth muscle cells

**FIGURE 2:**
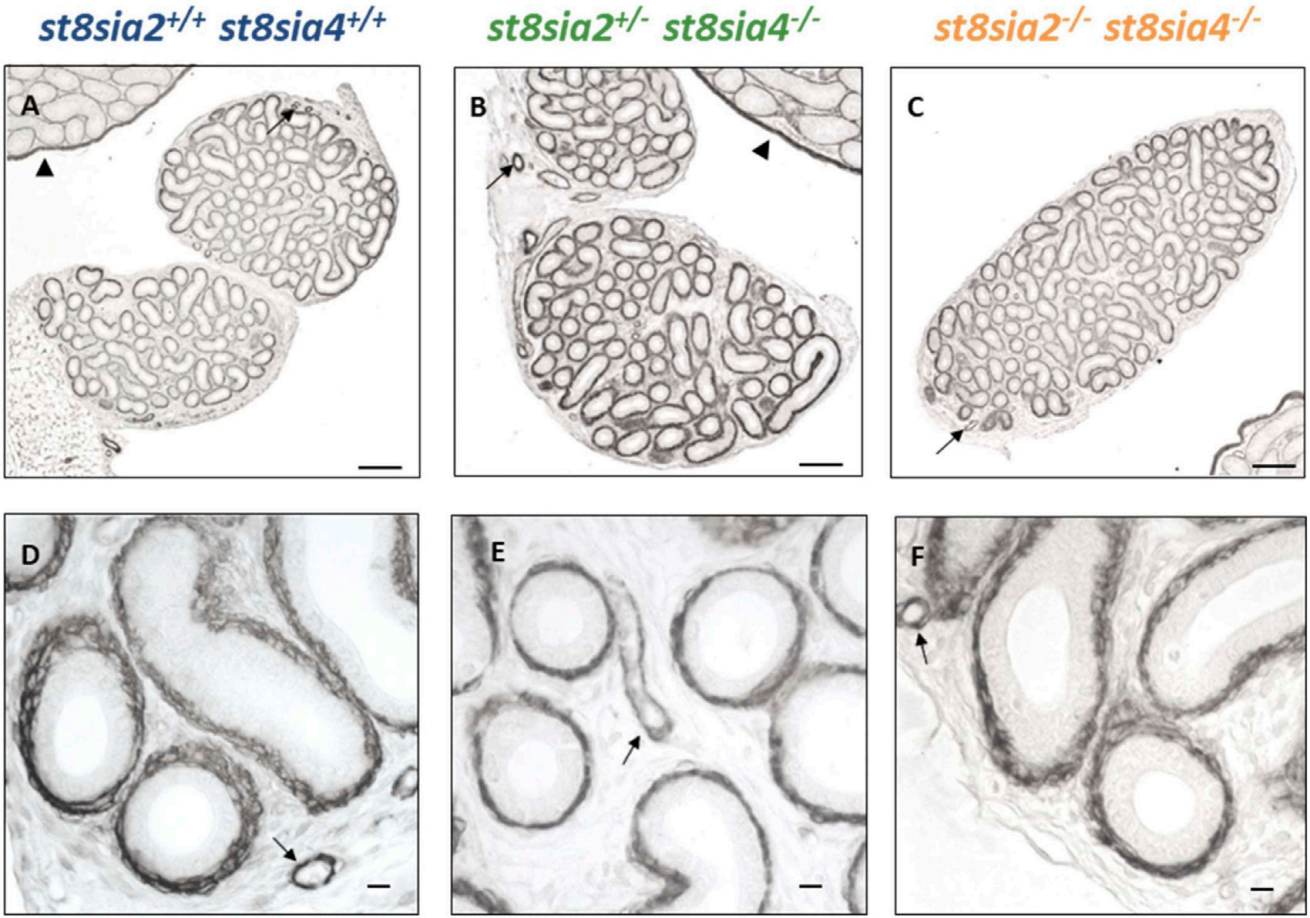
Localization of SMA in the postnatal epididymis of wild-type and knockout mice. Epididymal tissue sections of postnatal wild-type (*st8sia2* ^*+/+*^; *st8sia4* ^*+/+*^), *st8sia2* ^*+/−*^; *st8sia4* ^*−/−*^, and polysialyltransferase-deficient (*st8sia2* ^*−/−*^; *st8sia4* ^*−/−*^) mice were stained with an anti-SMA mAb. Arrows indicate blood vessels. **(A–C)** Testis is labeled with a triangle. Scale bars: **(A–C)** 100 μm; **(D–F)** 10 μm.

In the postnatal testis, the loss of polySia came along with a decrease of SMA in smooth muscle cells of seminiferous tubules (Hachem et al., 2021). For this reason, we visualized SMA in epididymal tissue of *st8sia2* ^*+/−*^; *st8sia4* ^*−/−*^ and *st8sia2* ^*−/−*^; *st8sia4* ^*−/−*^ mice and compared the immunostaining with wild-type mice (Figure 2). However, no discernible differences in SMA staining were detectable in the tubular system of the epididymis (i.e., epididymal duct) between the analyzed genotypes. In contrast, testicular tissue, visible in the same histological section (Figures 2A–C), showed the abovementioned differences in peritubular smooth muscle cells (Hachem et al., 2021). In accordance with the published data (Hachem et al., 2021), smooth muscle cells of blood vessels and tunica albuginea remained unchanged.

**FIGURE 3:**
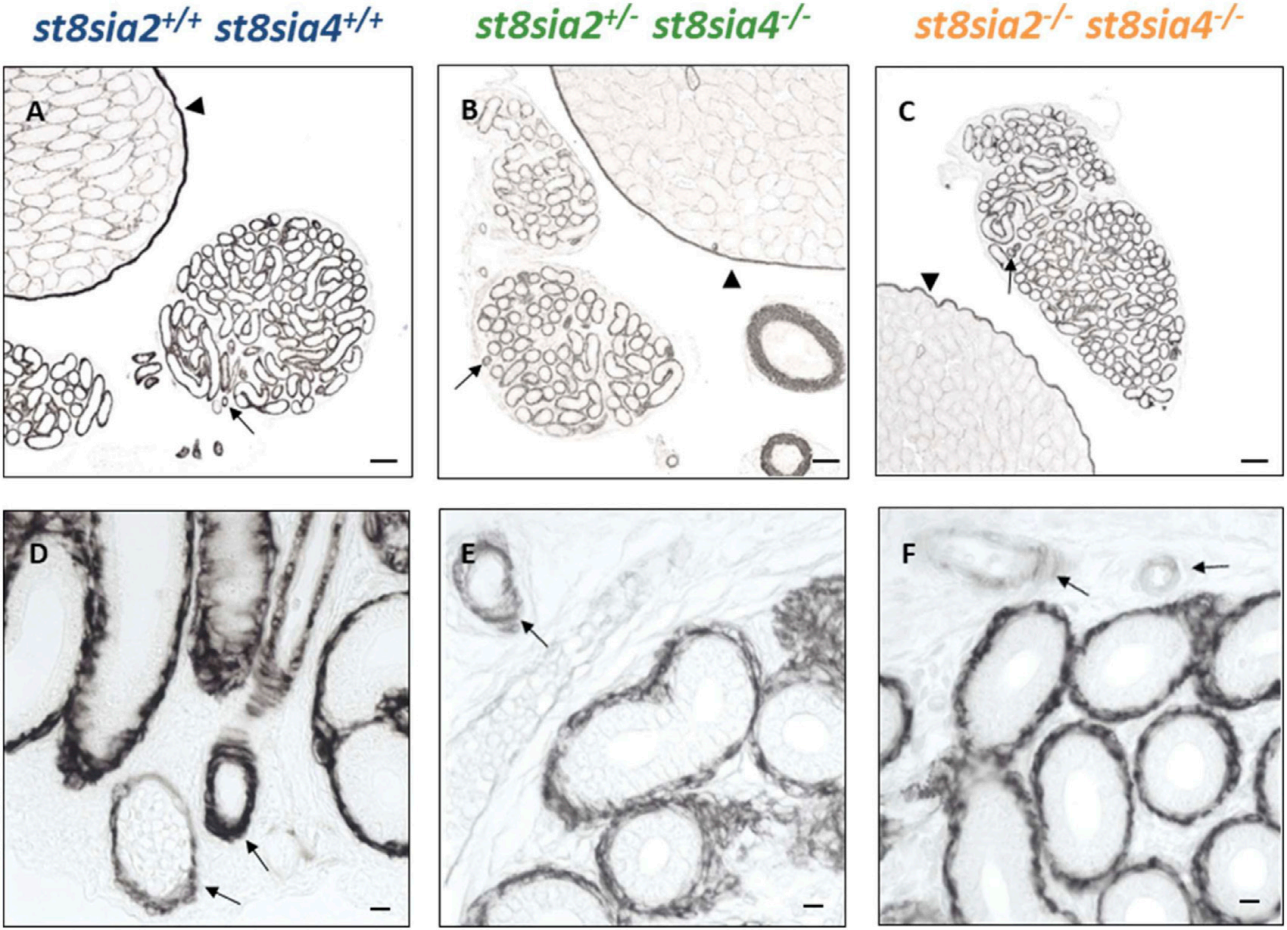
Distribution of calponinin in the postnatal epididymis of wild-type and knockout mice by immunostaining. For the immunohistochemical visualization of calponin in postnatal wild-type, *st8sia2* ^*+/−*^; *st8sia4* ^*−/−*^ and *st8sia2* ^*−/−*^; *st8sia4* ^*−/−*^ mice an mAb was applied. Arrows indicate blood vessels. **(A–C)** Testis is labeled with a triangle. Scale bars: **(A–C)** 100 μm; **(D–F)** 10 μm.

In addition to SMA, we analyzed the expression of another marker for muscle cells with calponin. Whereas SMA is present in all smooth muscle cells, the expression of calponin only takes place in more differentiated smooth muscle cells with a contractile phenotype (Ayoubi et al., 2017; Zhang et al., 2017). In the testes of polySia knockout mice (*st8sia2* ^*−/−*^; *st8sia4* ^*−/−*^), peritubular calponin staining was negative, different to smooth muscle cells of blood vessels and tunica albuginea (Figures 3A–C), which was already described in Hachem et al., 2021. However, such a difference between the genotypes was not detectable in the epididymal duct. All calponin immunostainings exhibited comparable signal intensities (Figure 3). Thus, we did not detect any influence of the loss of polySia on the expression of two important structural proteins of smooth muscle cells, SMA and calponin.

### 3.3 The expression of PKG1 is affected by the loss of polySia in smooth muscle cells of the epididymal duct

**FIGURE 4:**
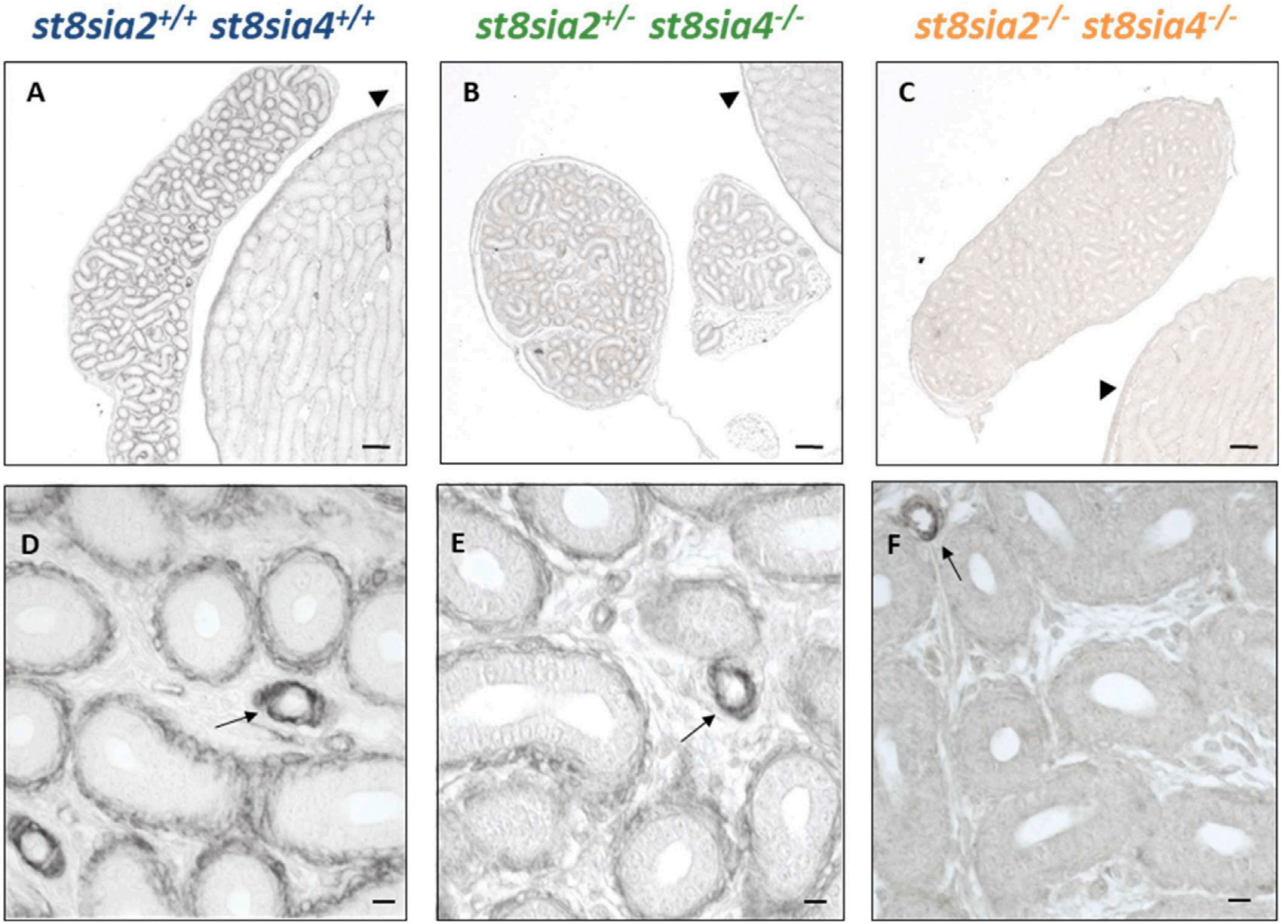
Localization of PKG1 in the postnatal epididymis of wild-type and knockout mice by immunostaining. PAbs were used for immunohistochemical visualization of PKG1 in epididymis of postnatal wild-type, *st8sia2* ^*+/−*^; *st8sia4* ^*−/−*^, and double knockout (*st8sia2* ^*−/−*^; *st8sia4* ^*−/−*^) mice. **(A–C)** The testis is labeled with a triangle. Arrows indicate blood vessels. Scale bars: **(A–C)** 100 μm; **(D–F)** 10 μm.

Besides SMA and calponin, PKG1 expression was investigated in the epididymis of all three genotypes. In postnatal testes of *st8sia2* ^*+/−*^; *st8sia4* ^*−/−*^ mice, reduced levels of PKG1 and in double knockout mice (*st8sia2* ^*−/−*^; *st8sia4* ^*−/−*^), a loss of PKG1 staining was described in seminiferous tubules (Figures 4A–C) (Hachem et al., 2021). These alterations also occurred in the epididymal duct (Figure 4). In comparison to *st8sia2* ^*+/−*^; *st8sia4* ^*−/−*^ mice, slightly stronger PKG1 signals were detected in wild-type. In polysialyltransferase-negative mice (*st8sia2* ^*−/−*^; *st8sia4* ^*−/−*^), no staining for PKG1 occurred in the tubules of the testis and epididymis (seminiferous tubules and epididymal duct). In contrast, no changes in PKG1 staining between the groups were observed in vascular smooth muscle cells (Figures 4D–F). Thus, only smooth muscle cell populations, which surround the epithelial layer, were affected by the loss of polySia. In sum, our analysis of the smooth muscle cells in the tubular structures of the testis (Hachem et al., 2021) and epididymis revealed an impaired differentiation status in polysialyltransferase knock out mice.

### 3.4 Enlarged rete testis in polysialyltransferase knockout mice

**FIGURE 5:**
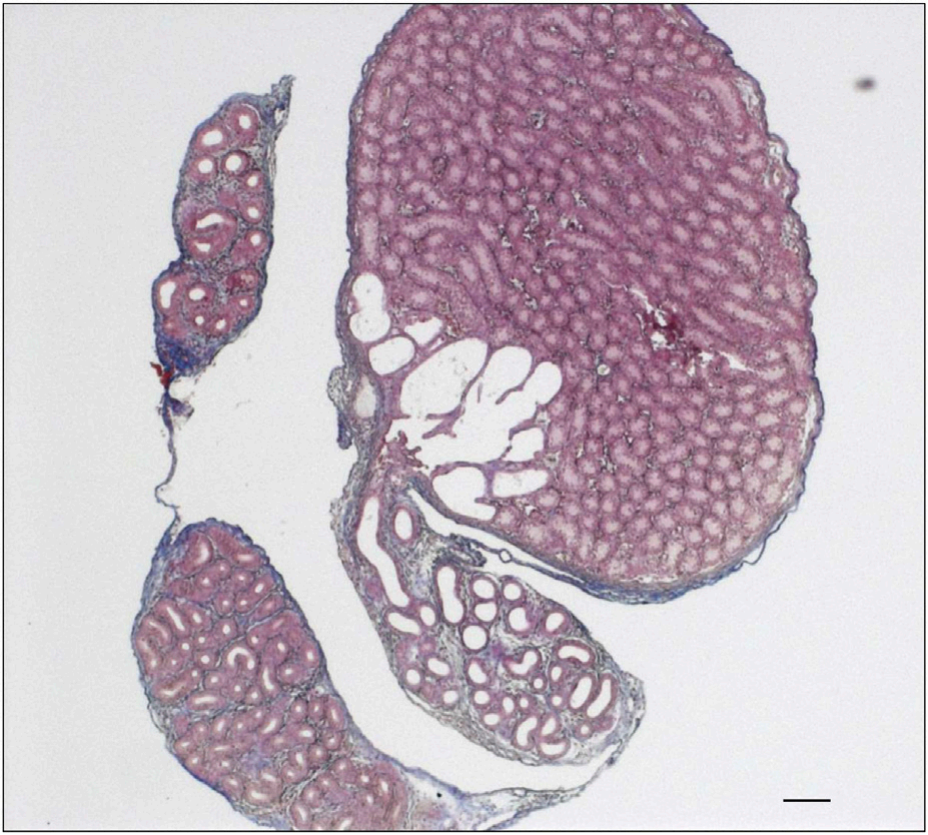
Rete testis of *st8sia2* ^*+/−*^ *; st8sia4* ^*−/−*^ mice. For the structural characterization of the epididymis and testis in *st8sia2* ^*+/−*^; *st8sia4* ^*−/−*^ mice, AZAN trichrome staining was used. Scale bar: 100 µm.

When investigating the connection between the testis and epididymis, namely, the rete testis, in routinely stained histological sections, surprisingly, a noticeable enlargement of the rete testis area was visible in *st8sia2* ^*+/−*^; *st8sia4* ^*−/−*^ mice (Figure 5). It is worth noting that in wild-type mice, some epithelial cells of the postnatal rete testis are polySia positive (Supplementary Figure S1).

**FIGURE 6:**
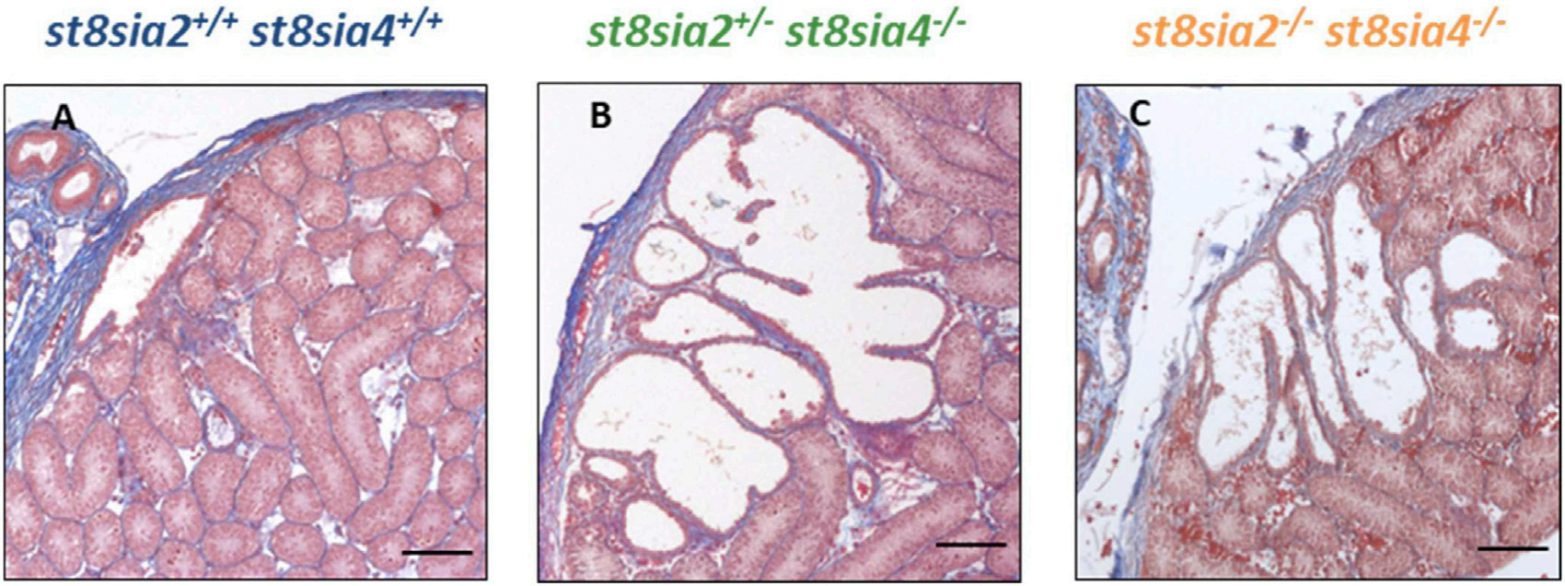
Rete testis of wild-type and knockout mice. For the structural characterization of rete testis, AZAN trichrome staining was used. **(A)** Wild-type (*st8sia2* ^*+/+*^; *st8sia4* ^*+/+*^), **(B)** *st8sia2* ^*+/−*^; *st8sia4* ^*−/−*^, and **(C)** polysialyltransferase-deficient (*st8sia2* ^*−/−*^; *st8sia4* ^*−/−*^) mice. Scale bar: 100 µm.

We systematically compared the rete testis of the outlined genotypes at the same age (Figure 6). In contrast to wild-type mice, *st8sia2* ^*+/−*^; *st8sia4* ^*−/−*^ and double knockout (*st8sia2* ^*−/−*^; *st8sia4* ^*−/−*^) mice showed an expanded rete testis, together with a widened luminal area.

## 4 Discussion

**FIGURE 7:**
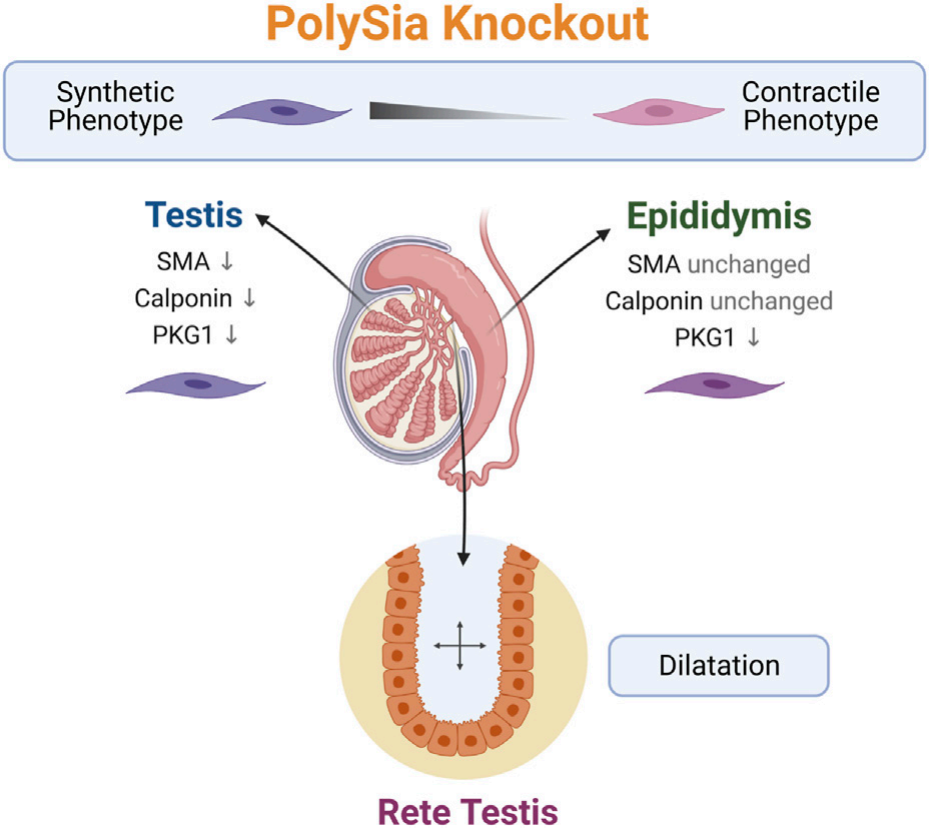
Summary of the observed phenotype in polySia knockout mice. The loss of polySia drives the differentiation of peritubular smooth muscle cells to a more synthetic phenotype and leads to a dilatation of rete testis. Created with BioRender.com.

During the postnatal development of the epididymis, the carbohydrate polymer polySia is localized in the smooth muscle cell layer of the epididymal duct (Simon et al., 2015). The presence of polySia is restricted to the first two postnatal weeks in mice. Simultaneously, the expression of both polysialyltransferases, ST8SiaII and ST8SiaIV, decreases. In adult animals, smooth muscle cells are polySia-negative. To determine the impact of polySia on epididymal development and function, we analyzed the postnatal epididymis of polysialyltransferase knockout mice. In both investigated genotypes, *st8sia2* ^*+/−*^; *st8sia4* ^*−/−*^ and *st8sia2* ^*−/−*^; *st8sia4* ^*−/−*^, no polysialylation of smooth muscle cells was observed. We used three markers to obtain information on smooth muscle cell differentiation. Not only the more general marker SMA but also calponin, indicating more differentiated smooth muscle cells, remained unchanged in the epididymal duct of knockout mice. In contrast to these structural proteins, the signaling molecule PKG1 was not detectable in smooth muscle cells of the epididymal duct when *st8sia2* and *st8sia4* were knocked out (Figure 7). In peritubular smooth muscle cells of the testicular duct system, a stronger effect on differentiation occurred (Hachem et al., 2021). Here, the expression of the investigated structural proteins was decreased. The impaired differentiation of the peritubular smooth muscle cells in the testis and epididymis suggests that the contraction in both organs is influenced.

Interestingly, the rete testis connecting the duct system of the testis with the epididymis and ensuring the transport of sperm and fluid was enlarged in both studied genotypes (*st8sia2* ^*+/−*^; *st8sia4* ^*−/−*^ and *st8sia2* ^*−/−*^; *st8sia4* ^*−/−*^) (Figure 7). Thus, a link between impaired polysialylation, a less differentiated smooth muscle cell phenotype and disturbed fluid flow is conceivable. A comparable morphological phenotype of the rete testis was observed when estrogen receptor α was knocked out (Hess et al., 1997; Lee et al., 2009). Already at postnatal day 10, dilatation of the rete testis was evident (Lee et al., 2009). In this model, a disturbed reabsorption of fluid by epithelial cells of the proximal epididymis was described (Hess et al., 1997). Our knockout model, however, is characterized by a disturbed contractile phenotype of peritubular smooth muscle cells in the testis (Hachem et al., 2021) and epididymis. Thus, smooth muscle cells of the duct system might essentially contribute to undisturbed anterograde fluid transport. It cannot be excluded that loss of polySia, found in some epithelial cells of the wild-type rete testis, may also contribute to changes in the rete testis.

A further interesting observation was that different to the epididymal duct, the differentiation of vascular smooth muscle cells was not affected by the loss of polySia. These results were in line with the described phenotype of testicular smooth muscle cells of polySia knockout mice (Hachem et al., 2021). The biochemical mechanisms for the different effects are unknown so far. PolySia can bind several growth factors, such as basic fibroblast growth factor (bFGF) or brain-derived neurotrophic factor (BDNF), and seems to influence their activation mechanisms (Kanato et al., 2008; Ono et al., 2012). Thus, the massive polysialylation of peritubular smooth muscle cells might play a role in the orchestra of growth factor action. In this context, it is of interest that no polySia immunostaining of vascular smooth muscle cells was detected in the epididymis.

In summary, our results demonstrate that the loss of polySia is accompanied by a less differentiated phenotype of smooth muscle cells surrounding the epithelium of the epididymal duct. The effects on the contractile system might influence fluid transport in the duct, resulting in the observed enlargement of the rete testis.

## Data Availability

The original contributions presented in the study are included in the article/Supplementary Material, further inquiries can be directed to the corresponding authors.
