# Supplementary material for: Knockout of the polysialyltransferases ST8SiaII and ST8SiaIV leads to a dilatation of rete testis during postnatal development
Source: Front Physiol. 2023 Jul 14;14:1240296. doi: 10.3389/fphys.2023.1240296 (PMC10382229; doi:10.3389/fphys.2023.1240296)
Supplement: Supplementary file 1 [file DataSheet1.PDF]

Supplementary Material

Knockout of the Polysialyltransferases ST8SiaII and ST8SiaIV Leads to a Dilatation of Rete Testis during Postnatal Development

Luisa Humpfle[1,¶], Nadim E. Hachem[1,¶], Peter Simon[1,2], Birgit Weinhold[3], Sebastian P. Galuska[4,*] and Ralf Middendorff[1,*]

*** Correspondence:**

Sebastian P. Galuska

galuska.sebastian@fbn-dummerstorf.de

Ralf Middendorff

ralf.middendorff@anatomie.med.uni-giessen.de

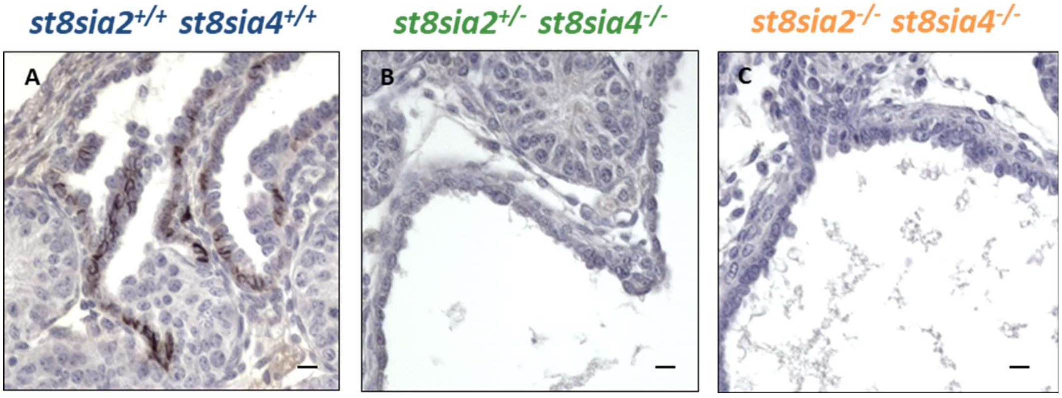

Supplementary Figure 1. Visualization of polySia in postnatal rete testes of 9-day-old wild-type and knockout mice. For the immunohistochemical localization of polySia in wild-type (*st8sia2*$^{+/+}$; *st8sia4*$^{+/+}$), *st8sia2*$^{+/-}$; *st8sia4*$^{-/-}$, and polysialyltransferase-deficient (*st8sia2*$^{-/-}$; *st8sia4*$^{-/-}$) mice, mAb 735 was applied. Nuclei were stained with hematoxylin. All scale bars: 10 µm.
